# Testicular organoid models in male reproductive toxicology: advances, applications, and future perspectives

**DOI:** 10.3389/fphys.2026.1874148

**Published:** 2026-07-30

**Authors:** Yan Liu, Yuting Sun, Meina Xie

**Affiliations:** School of Life Science and Technology, Shandong Second Medical University, Weifang, Shandong, China

**Keywords:** blood-testis barrier, endocrine-disrupting chemicals, male reproductive toxicology, organ-on-chip, spermatogenesis, testicular organoid

## Abstract

Male reproductive health has declined markedly over the past decades, with environmental chemicals, pharmaceuticals, and lifestyle factors implicated in this trend. Conventional toxicological approaches—rodent bioassays and two-dimensional cell cultures—suffer from species-specific biases, limited physiological relevance, and inadequate throughput for screening the expanding universe of suspect compounds. Testicular organoid technology has emerged as a promising alternative, reconstituting three-dimensional cellular architecture, paracrine crosstalk, and partial spermatogenic progression *in vitro*. This narrative review was assembled from English-language studies retrieved from PubMed, Web of Science, and Scopus (2010–2024) using combinations of the terms testicular organoid, organ-on-chip, spermatogenesis, and reproductive toxicology, prioritizing original research and methodological reports on three-dimensional testicular models and excluding abstract-only records. On this basis the review maps current construction strategies, spanning primary cell isolation, pluripotent stem cell differentiation, and spermatogonial stem cell expansion, alongside matrix choices and microfluidic culture platforms. It then examines how testicular organoids have been deployed to assess endocrine-disrupting chemicals, chemotherapeutics, and other pharmacological agents, where several studies report the detection of effects at concentrations appreciably below those required in monolayer systems, particularly under chronic low-dose exposure. Integration with single-cell multi-omics and machine learning has advanced mechanistic dissection and candidate biomarker discovery across oxidative, mitochondrial, and epigenetic axes, although these computational applications remain exploratory given the absence of standardized organoid protocols. Morphological, functional, molecular, and epigenetic indicators are consolidated into a unified evaluation framework, and persistent bottlenecks are addressed, including incomplete blood-testis barrier reconstitution, long-term culture instability, inter-donor variability, and inefficient elongated spermatozoa formation. Overall, testicular organoids offer clear mechanistic and human-relevance advantages, yet they are not yet sufficiently mature or validated to broadly replace *in vivo* models in regulatory assessment. Future priorities center on protocol standardization, automated high-throughput adaptation, multi-organ-on-chip integration, and formal regulatory validation to enable transformative applications in drug safety evaluation, environmental risk management, and male reproductive health protection.

## 1 Introduction

Male reproductive health has deteriorated at an alarming pace over the past several decades, emerging as a pressing public health concern that cuts across industrialized and developing nations alike. A landmark meta-analysis drawing on 244 estimates from 7,518 men across 53 countries documented a 51.6% decline in sperm concentration and a 62.3% reduction in total sperm count between 1973 and 2018, with the slope of decline accelerating after the year 2000 (Levine et al., 2023). Parallel epidemiological surveillance has flagged rising incidences of testicular dysgenesis syndrome, cryptorchidism, and testicular germ cell tumors in young men, pointing to insults operating during fetal programming windows (Sharpe, 2020). Environmental chemical exposure—spanning phthalates, bisphenols, perfluoroalkyl substances, pesticide residues, and heavy metals—has been consistently linked to these trends through both human cohort data and mechanistic investigations (Gore et al., 2020). The biological plausibility is compelling: many of these compounds disrupt androgen signaling, perturb Sertoli–germ cell crosstalk, or induce oxidative damage within the seminiferous epithelium (Cheng and Mruk, 2012). Yet causal attribution at the individual compound level remains elusive, largely because our assessment toolkit lags behind the complexity of real-world exposures.

Traditional reproductive toxicology has leaned heavily on rodent bioassays, which, while informative, suffer from a stubborn species-gap problem. Murine spermatogenesis unfolds over roughly 35 days versus approximately 74 days in humans, and the transcriptional choreography of spermatogonial stem cell maintenance diverges markedly between species (Sobinoff et al., 2021). Compounds such as 2,5-hexanedione and certain glycol ethers exhibit dramatically different testicular toxicity profiles in rats than in primates or humans, leading to both false positives and missed hazards when rodent data are extrapolated to human risk assessment (Foster, 2017). Ethical pressure, encoded in the 3Rs principle and formalized through regulatory initiatives like REACH and the U.S. EPA’s push toward new approach methodologies, has further constrained the scope of permissible *in vivo* testing. Two-dimensional primary testicular cell cultures were developed as a partial remedy, yet they fail to preserve the blood-testis barrier, lose Leydig cell steroidogenic competence within days, and cannot sustain meiotic progression—meaning that the very endpoints most relevant to human fertility simply cannot be read out (Schlatt and Ehmcke, 2014).

Testicular organoid technology has moved into this gap. Early work by Baert and colleagues demonstrated that dissociated human testicular cells could self-organize into tubule-like structures retaining Sertoli cell polarity and tight junction formation (Baert et al., 2015). Subsequent efforts using rodent models achieved, for the first time, *in vitro* meiosis and limited haploid cell production within three-dimensional constructs (Sato et al., 2011). Chinese research groups have contributed substantially through hydrogel optimization and the incorporation of induced pluripotent stem cell-derived germ cell precursors, broadening the donor base beyond biopsy-dependent primary cells (Zhang et al., 2014). Despite these advances, three challenges continue to constrain the field. Construction protocols differ considerably between laboratories—varying in cell ratios, matrix composition, culture duration, and hormonal supplementation—which complicates direct cross-study comparison, a limitation examined further in the Discussion. Functional maturity also remains incomplete; most reported organoids plateau at pachytene or round spermatid stages, with elongated spermatozoa rarely observed. And, perhaps most importantly, a coherent toxicological evaluation framework tailored to organoid-specific readouts has yet to be codified, leaving investigators to borrow *ad hoc* endpoints from conventional assays (Alves-Lopes and Stukenborg, 2018).

Organoid-based reproductive toxicology represents more than a matter of animal replacement. It offers a route toward mechanistic resolution that neither rodent studies nor flat cultures can deliver, and it aligns with the broader pivot toward precision toxicology, where chemical effects are interpreted against the backdrop of human-relevant cellular architecture (Griswold, 2016). To map this landscape, the present narrative review draws on English-language literature retrieved from PubMed, Web of Science, and Scopus (2010–2024) using combinations of the search terms testicular organoid, organ-on-chip, spermatogenesis, endocrine-disrupting chemical, and reproductive toxicology; original studies and methodological reports describing three-dimensional testicular models were prioritized, whereas conference abstracts and non-English records were excluded. On this basis the review maps current construction strategies for testicular organoids, dissects their deployment within toxicological evaluation pipelines, and benchmarks their predictive performance against legacy approaches. Its intended contribution is the integration of multidimensional assessment indicators—morphological, molecular, functional, and epigenetic—into a unified framework for organoid-based reproductive toxicology, which the existing literature has not yet attempted in consolidated form.

## 2 Testicular physiology and the foundations of male reproductive toxicology

### 2.1 Histological architecture of the testis and the mechanism of spermatogenesis

The mammalian testis operates as a dual-compartment organ, partitioned into the tubular compartment where germ cell maturation unfolds and the interstitial compartment housing the endocrine machinery that fuels it. Seminiferous tubules, coiled into dense loops within each testicular lobule, are lined by a stratified germinal epithelium embedded in a basement membrane reinforced by peritubular myoid cells whose rhythmic contractions propel spermatozoa toward the rete testis (Sharpe, 2015). Within the interstitium, Leydig cells cluster near capillaries and lymphatic sinusoids, synthesizing testosterone under pulsatile luteinizing hormone stimulation and sustaining the high intratesticular androgen concentrations that the germinal epithelium depends upon (Hedger and de Kretser, 2020). Separating these two worlds is the blood-testis barrier, a junctional specialization formed between adjacent Sertoli cells through coordinated tight junctions, ectoplasmic specializations, desmosomes, and gap junctions, which cordons off post-meiotic germ cells from systemic immune surveillance while enforcing a selective biochemical milieu (Yan et al., 2008).

Spermatogenesis itself proceeds as a meticulously timed relay. Spermatogonial stem cells residing on the basal lamina balance self-renewal with commitment to differentiation, giving rise to type A and type B spermatogonia that ultimately enter meiosis as primary spermatocytes (de Rooij, 2017). Two successive meiotic divisions produce haploid round spermatids, which then undergo spermiogenesis—a profound morphogenetic remodeling involving acrosome biogenesis, nuclear condensation via histone-to-protamine replacement, flagellar assembly, and cytoplasmic shedding—before release into the tubular lumen as elongated spermatozoa. The entire cascade is organized into spatially fixed epithelial stages, and a single cycle spans roughly 74 days in humans, a timeline that any faithful *in vitro* system must ideally reproduce.

What makes this process tractable, and simultaneously fragile under toxicant exposure, is the dense paracrine dialogue among its cellular participants. Sertoli cells secrete stem cell factor, glial cell line-derived neurotrophic factor, and activin to nurture spermatogonia, while receiving androgen signals from neighboring Leydig cells that they relay forward through androgen receptor-dependent transcriptional programs (Mäkelä et al., 2019). Germ cells reciprocate by releasing factors that modulate Sertoli cell metabolism and barrier dynamics, and peritubular myoid cells contribute extracellular matrix components and retinoic acid precursors that further tune the niche. Disruption of any single node—whether by xenobiotic-induced oxidative stress, receptor antagonism, or junctional destabilization—can cascade through the network and arrest spermatogenesis at characteristic stages. **Table 1** summarizes the principal cell populations, their spatial niches, and the functional outputs most relevant to toxicological readouts. As presented in **Table 1**, the functional heterogeneity of testicular cells explains why no single-cell-type assay can capture the full spectrum of reproductive toxicity, and why three-dimensional reconstitution of this cellular ecology has become such a central pursuit (Potter and Defalco, 2017). In practical terms, this physiology defines the minimum requirements that a testicular organoid must satisfy to be considered physiologically relevant: a functional spermatogonial niche, clear basal-to-adluminal compartmentalization, dynamic blood-testis barrier formation, and at least partial temporal synchronization of spermatogenesis.

**Table 1:** Principal cell types of the testis and their physiological functions.

Cell type	Anatomical location	Principal function	Key markers
Spermatogonial stem cells	Basal compartment of seminiferous tubule	Self-renewal and generation of differentiating progeny	PLZF, GFRα1, ID4
Sertoli cells	Seminiferous epithelium, spanning basal to adluminal	Nursing of germ cells; blood-testis barrier formation	SOX9, WT1, GATA4
Leydig cells	Interstitial stroma adjacent to capillaries	Testosterone biosynthesis under LH control	CYP17A1, StAR, INSL3
Peritubular myoid cells	Surrounding seminiferous tubule basement membrane	Contractile transport of spermatozoa; matrix deposition	α-SMA, Desmin
Spermatocytes and spermatids	Adluminal compartment	Meiotic division and spermiogenic morphogenesis	SYCP3, Protamine 1/2
Testicular macrophages	Interstitial stroma	Immune surveillance; Leydig cell trophic support	F4/80, CD68

### 2.2 Classification of male reproductive toxicants and their molecular targets

Environmental male reproductive toxicants are a heterogeneous collection rather than a coherent chemical family, and any workable classification must reconcile chemical identity with biological action. By source and structure, five broad categories dominate contemporary concern. Endocrine-disrupting chemicals—including phthalate esters, bisphenol A and its structural analogs, parabens, and polybrominated diphenyl ethers—interfere with steroid hormone synthesis, transport, or receptor engagement, often at concentrations well below classical toxicity thresholds (Joensen et al., 2020). Heavy metals such as cadmium, lead, mercury, and arsenic accumulate in testicular tissue through divalent cation transporters and inflict damage through oxidative stress, protein thiol depletion, and blood-testis barrier disassembly (Rzymski et al., 2015). Pesticide residues, notably organophosphates, organochlorines, and neonicotinoids, reach the gonad via dietary and occupational routes and exhibit mixed mechanisms encompassing cholinesterase inhibition and AhR-mediated transcriptional perturbation (Sifakis et al., 2017). A growing catalog of pharmaceuticals—alkylating chemotherapeutics, certain antiepileptics, and select antihypertensives—produces iatrogenic spermatotoxicity that is sometimes irreversible. Lifestyle-linked exposures, including tobacco combustion products, ethanol, cannabinoid constituents, and chronic heat stress from occupational sources, round out the picture and frequently act as co-exposures that amplify chemical insults. Several emerging contaminant classes warrant particular attention given the breadth of male reproductive concern. Per- and polyfluoroalkyl substances (PFAS) are persistent, bioaccumulative compounds for which epidemiological and experimental data have linked exposure to altered steroidogenesis, reduced semen quality, and disrupted hypothalamic-pituitary-gonadal signaling (Fenton et al., 2021). Microplastics and nanoplastics, now detected in human tissues, can carry adsorbed pollutants and have been associated with testicular oxidative stress and blood-testis barrier perturbation (Vethaak and Legler, 2021). Engineered nanomaterials raise analogous concerns, as their small size favors testicular accumulation and reactive-oxygen-species generation. Critically, real-world exposure involves complex mixtures rather than single agents, and combined exposures can produce additive or synergistic effects that single-compound assessment systematically underestimates (Drakvik et al., 2020). These features make multicellular, human-relevant *in vitro* models especially attractive for capturing the low-dose and mixture effects that conventional assays tend to overlook.

Mapping these agents onto cellular and axis-level targets clarifies why their phenotypic footprints differ so markedly. At the germ cell tier, alkylators and certain metals provoke DNA double-strand breaks preferentially during meiotic prophase, when homologous recombination machinery is already loaded onto chromatin and particularly vulnerable to additional lesions (Dobrzyńska and Radzikowska, 2013). Somatic cells are hit along distinct axes: Sertoli cells lose barrier integrity when junctional adhesion proteins such as occludin and connexin 43 are destabilized by phthalate metabolites, whereas Leydig cells respond to cadmium and bisphenol analogs with mitochondrial dysfunction and steroidogenic enzyme suppression, primarily targeting StAR and CYP17A1 (Siu et al., 2009). At the systemic level, the hypothalamic-pituitary-gonadal axis itself becomes a target: xenoestrogens feedback on GnRH pulsatility, while anti-androgens blunt peripheral androgen-responsive transcription, producing dysregulation that cannot be captured by isolated cell assays (Patisaul, 2017). This tiered vulnerability explains a recurring observation in the literature: organoid systems that retain multiple cell types often reveal toxicities which monoculture screens miss entirely.

Quantitative description of these responses relies on dose-response modeling. The classical Hill equation, long standard in pharmacology, captures sigmoidal toxicity curves through (Equation 1).

(1) $$E=E_{max}\cdot \frac{C^{n}}{EC_{50}^{n}+C^{n}}$$

where *E* denotes the observed effect, *C* the exposure concentration, *EC*_50_ the half-maximal effective concentration, and *n* the Hill coefficient reflecting cooperativity (Goutelle et al., 2008). Non-monotonic responses, common among endocrine disruptors, defeat this framework and are better represented by a biphasic hormesis function such as the Brain-Cousens model (Equation 2):

(2) $$E=c+\frac{d-c+f\cdot C}{1+exp\left[b\cdot \left(lnC-lnEC_{50}\right)\right]}$$

in which *f* parameterizes the low-dose stimulatory phase and *b*, *c*, *d* the descending branch parameters (standard formulation). Choosing between these models is not a cosmetic decision; it determines whether a compound is flagged as safe at environmentally relevant concentrations or identified as a low-dose hazard.

### 2.3 Conventional approaches in male reproductive toxicology and their inherent limitations

Regulatory toxicology has long rested on a scaffolding of standardized *in vivo* assays. The OECD Test Guidelines—chiefly TG 407 (28-day repeated dose), TG 408 (90-day subchronic), TG 416 (two-generation reproductive study), and the extended one-generation protocol TG 443—prescribe rodent dosing regimens, histopathological scoring of the testis and epididymis, sperm count and motility analysis, and assessment of F1 fertility endpoints (Knudsen et al., 2020). Rat and mouse models remain the workhorses of these protocols, chosen for their tractable breeding cycles and deep historical control databases, yet the very uniformity that gives regulatory data their comparability also obscures interspecies divergence at the tissue level. Cost is another stubborn factor: a single TG 443 study consumes roughly two years and several hundred animals, which rules out systematic screening of the tens of thousands of chemicals now in commercial circulation (Hartung, 2009).

*In vitro* alternatives sit on the other end of the trade-off. Immortalized Sertoli lines such as TM4 and 15P-1, Leydig lines including TM3 and MA-10, and primary co-cultures in serum-supplemented monolayers have been the default for mechanistic dissection since the 1980s (Mruk and Cheng, 2015). Ex vivo testicular tissue slices and seminiferous tubule segments preserve short-term architecture and can sustain partial spermatogenesis for one to two weeks under gas-liquid interface conditions, offering a middle ground between flat cultures and whole-animal work (Reda et al., 2014). But monolayers shed their polarity within passages, lose stage-specific gene expression, and cannot recapitulate the blood-testis barrier in any functionally meaningful way; tissue slices, though architecturally richer, suffer from donor variability, necrotic cores, and throughput ceilings that make them unsuitable for dose-response mapping across large compound libraries.

Several inherent limitations cut across these methods. Species differences distort human risk extrapolation, as mentioned earlier—glycol ethers and phthalate monoesters produce potency shifts of one to two orders of magnitude between rodent and primate testicular cells (Gray et al., 2022). Physiological relevance is compromised whenever three-dimensional architecture, hormonal pulsatility, or multi-lineage crosstalk is absent from the assay, which is almost always the case for monocultures. Throughput remains mismatched with regulatory demand, and ethical pressure under the 3Rs framework has intensified the push for replacement technologies beyond cosmetic refinements (Balls, 2020). Against this backdrop, the emergence of testicular organoid models reads less as a technological novelty than as a corrective response to gaps the field has acknowledged for years—a convergence of stem cell biology, microengineering, and regulatory need.

## 3 Construction strategies and key technologies for testicular organoid models

### 3.1 Cellular sources and culture systems for testicular organoid construction

The choice of starting cellular material shapes nearly every downstream property of a testicular organoid, from its maturation ceiling to its suitability for toxicological interrogation. Three principal sourcing strategies have crystallized in the field, each with distinct practical trade-offs. Primary testicular cell isolation, typically performed through two-step enzymatic digestion of biopsy tissue with collagenase IV followed by trypsin-DNase treatment, yields mixed populations containing Sertoli, Leydig, peritubular myoid, and germ cells in ratios that roughly mirror the donor tissue (de Michele et al., 2017). This approach preserves native cellular identity and has produced the most functionally mature organoids reported to date, yet it remains constrained by donor scarcity, inter-individual heterogeneity, and the ethical sensitivities surrounding human testicular biopsies. Pluripotent stem cell-based strategies offer a scalable alternative: human induced pluripotent stem cells can be directed through a primordial germ cell-like cell intermediate using BMP4, SCF, and LIF cocktails, then co-cultured with somatic support cells to assemble testicular structures (Sakib et al., 2019a). Spermatogonial stem cell expansion represents a third route, exploiting the self-renewal capacity of GFRα1-positive cells cultured on mitotically arrested feeders in the presence of GDNF and FGF2, with reported expansion factors exceeding 10^4^ over several months (Kubota and Brinster, 2018). The relative merits of these sources deserve a more critical reading. Immortalized lines afford reproducibility and scale but carry transformed phenotypes that distort steroidogenic and junctional behavior; adult human cells best preserve mature function yet are scarce and difficult to expand; prepubertal tissues are more proliferative but have not completed the developmental programs that toxicologically relevant endpoints depend upon. Rodent and other animal-derived models remain valuable for proof-of-concept and benchmarking, but interspecies differences in spermatogenic timing, hormonal regulation, and xenobiotic metabolism limit their predictive transfer to human risk and reinforce the case for human-derived systems wherever feasible.

Three-dimensional matrices translate dissociated cells into organized structures. Matrigel, the basement membrane extract from Engelbreth-Holm-Swarm sarcoma, remains the default scaffold owing to its laminin- and collagen IV-rich composition that promotes tubule-like self-organization within 48 to 72 hours, though batch variability and xenogenic origin limit its translational appeal (Alves-Lopes et al., 2017). Decellularized testicular extracellular matrix, prepared through sequential detergent treatment and DNA removal, retains tissue-specific protein signatures including testicular laminin isoforms and growth factor reservoirs, and has been shown to extend germ cell survival relative to generic matrices (Vermeulen et al., 2018). Synthetic and semi-synthetic hydrogels—polyethylene glycol-based networks, alginate-collagen composites, and gelatin methacryloyl formulations—offer tunable stiffness in the 0.5 to 5 kPa range matching native testicular mechanics and permit precise control over degradation kinetics (Richer et al., 2020).

**Figure 1:**
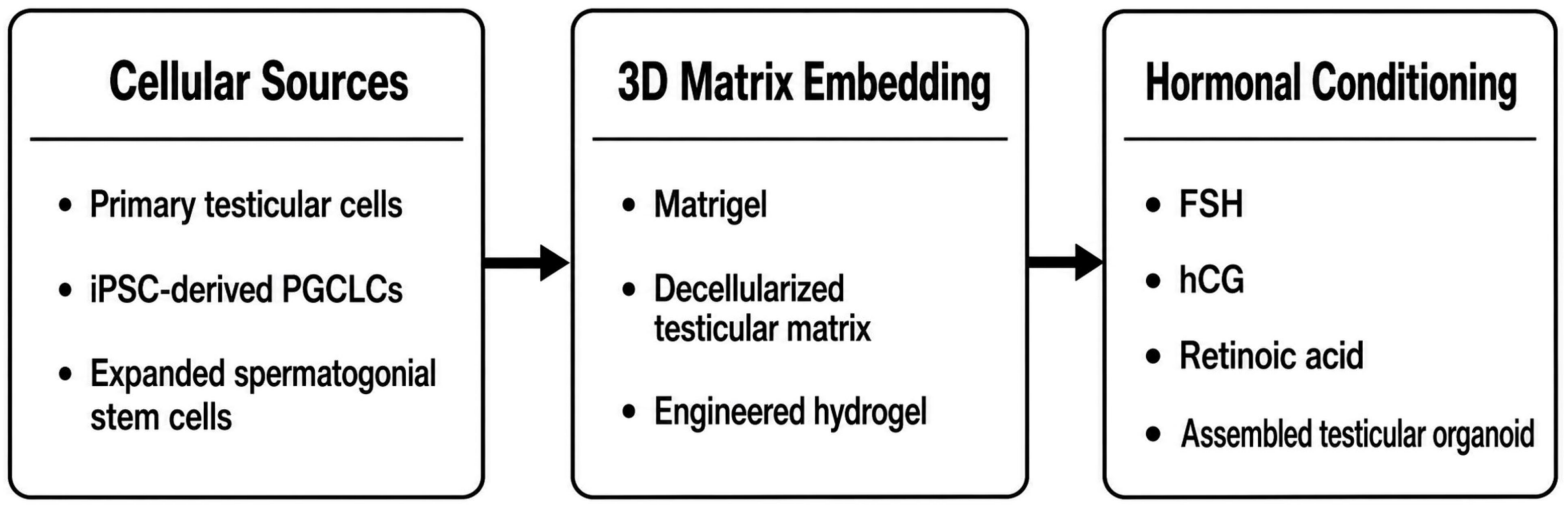
Conceptual schematic prepared by the authors, summarizing testicular organoid construction strategies: parallel routes from primary cells, pluripotent stem cell derivatives, and expanded spermatogonial stem cells, followed by three-dimensional embedding in Matrigel, decellularized matrix, or engineered hydrogels under defined hormonal stimulation. The figure is an integrative illustration rather than a representation of a single published dataset.

Growth factor and hormone supplementation orchestrates developmental progression within these scaffolds. Follicle-stimulating hormone at 10–100 mIU/mL drives Sertoli cell maturation and androgen-binding protein secretion, while human chorionic gonadotropin sustains Leydig cell testosterone output at concentrations approaching physiological intratesticular levels. Retinoic acid pulses at 1 μM have proven essential for triggering meiotic entry, mirroring the *in vivo* role of somatic retinoid signaling (Griswold et al., 2012). Kit ligand, GDNF, and activin A are commonly layered into defined media to preserve spermatogonial niche integrity during extended culture (Kanatsu-Shinohara and Shinohara, 2013). **Figure 1** outlines the integrated construction pipeline, from cellular sourcing through matrix embedding to hormonal conditioning.

**Table 2** compares these sourcing strategies across maturation endpoints and practical constraints.

**Table 2:** Comparison of testicular organoid construction methods by cellular source.

Cell source	Maturation endpoint	Scalability	Inter-batch consistency	Primary applications
Primary testicular cells	Round spermatid to elongated	Low	Moderate	Mechanistic toxicology, fertility research
iPSC-derived PGCLCs	Pachytene spermatocyte	High	High	High-throughput screening, disease modeling
Expanded SSCs	Round spermatid	Moderate-high	Moderate	Niche studies, genotoxicity assays
Rodent primary cells	Elongated spermatid	Moderate	High	Proof-of-concept, protocol benchmarking
Mixed xenogeneic co-culture	Pachytene	Moderate	Low	Exploratory studies only

As **Table 2** indicates, the tension between maturation depth and scalability remains unresolved, and the choice of source must be aligned with the specific toxicological question at hand.

### 3.2 Culture platforms and organ-on-chip technologies for testicular organoids

Beyond cellular sourcing and matrix selection, the physical culture platform exerts its own decisive influence on organoid fate. Three conventional approaches dominate the benchtop landscape, each operating on distinct biophysical principles. Hanging drop culture, in which 20–40 μL droplets containing dissociated cells are suspended from inverted lids, exploits surface tension to force cell–cell contact at the air-liquid meniscus, generating compact spheroids within 24 to 48 hours (Baert et al., 2017). The method is elegantly simple and well suited to small-scale spheroid generation, though its open geometry limits long-term feeding and precludes precise control over oxygen tension. Gas-liquid interface culture, popularized by the Sato laboratory for murine testicular tissue, positions tissue fragments atop agarose stands partially submerged in medium, allowing apical oxygen exchange while basal nutrients diffuse upward (Sato et al., 2013). This configuration has sustained meiotic completion in neonatal mouse explants for up to two months and remains the most reliable platform for rodent spermatogenesis recapitulation. Stirred and rotating-wall bioreactors offer a third option, reducing shear while maintaining continuous medium perfusion, which improves scalability but introduces mechanical stress that can destabilize delicate junctional complexes within assembling organoids (Lee et al., 2022).

**Figure 2:**
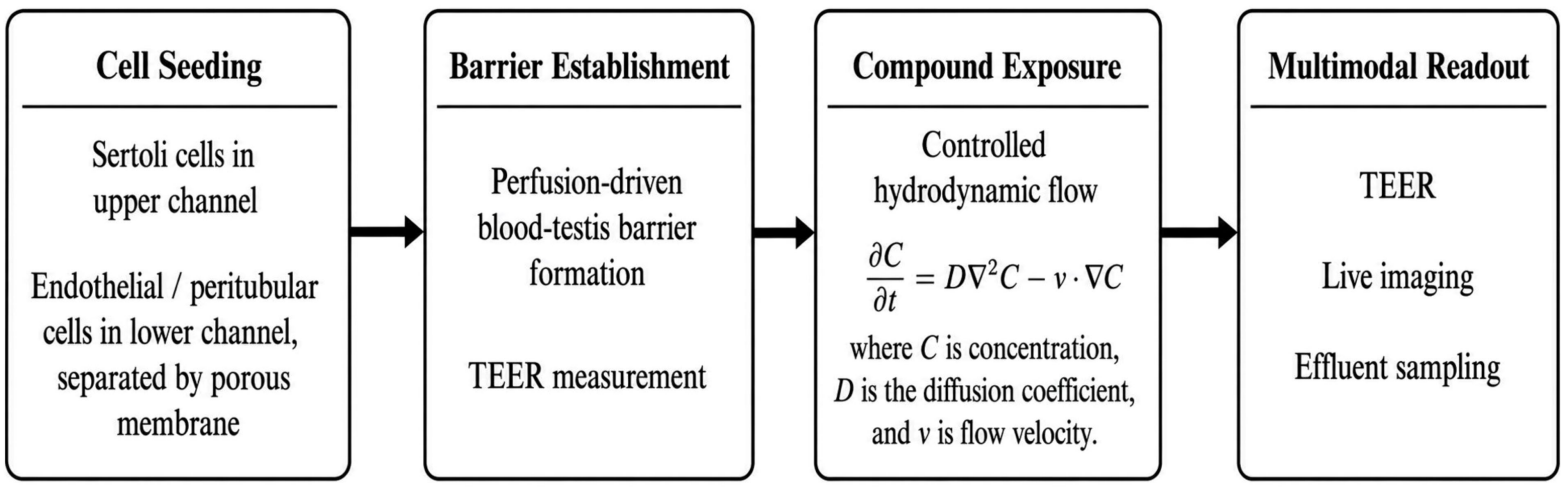
Conceptual workflow of a testicular organ-on-chip platform prepared by the authors, depicting sequential cell seeding into compartmentalized channels, perfusion-driven establishment of blood-testis barrier function, coupled exposure to test compounds under controlled hydrodynamic conditions, and multimodal readouts including TEER, live imaging, and effluent sampling for downstream analysis. It is an integrative schematic rather than a depiction of a specific published dataset.

Microfluidic organ-on-chip platforms differ conceptually from organoids and merit a brief definition. Whereas an organoid is a self-organizing three-dimensional cell aggregate, an organ-on-chip is an engineered microfluidic device that imposes defined fluid flow, chemical gradients, and physical compartmentalization on cells or on embedded organoids; the two are complementary and increasingly combined, but they are distinct platforms and are not treated as synonyms here. Such devices push beyond static paradigms by embedding dynamic physicochemical gradients directly into the culture architecture. Blood-testis barrier-on-chip devices typically employ two parallel channels separated by a porous polycarbonate or PDMS membrane, with Sertoli cells seeded on one side and endothelial or peritubular cells on the other, enabling real-time measurement of transepithelial electrical resistance as a quantitative barrier readout (Park et al., 2021). Such designs have reproduced cadmium-induced barrier disruption at concentrations an order of magnitude below those required in static cultures, a sensitivity gain of particular relevance for low-dose toxicant screening. Multi-organ interconnected chips extend this logic by linking testicular compartments with hepatic and renal modules through shared perfusion loops, thereby incorporating first-pass metabolism and excretion into the exposure pipeline—an arrangement that captures, for instance, the bioactivation of di(2-ethylhexyl) phthalate to its more potent monoester metabolite before it reaches the gonadal compartment (Skardal et al., 2017). Mass transport within these systems follows the convection-diffusion equation governing xenobiotic distribution along the microchannel (Equation 3):

(3) $$\frac{\partial C}{\partial t}=D\nabla^{2}C-v\cdot \nabla C$$

where *C* is the local concentration, *D* the diffusion coefficient, and *v* the flow velocity vector (standard formulation). **Figure 2** outlines the integrated workflow from cell loading through perfusion-coupled toxicant exposure to multi-parametric readout.

Bioprinting has emerged as a complementary route for reconstructing spatial heterogeneity that neither self-organization nor membrane partitioning can fully deliver. In essence, bioprinting deposits cell-laden hydrogel “bioinks” in pre-programmed patterns to build tissue constructs with defined geometry. Extrusion-based deposition of Sertoli-germ cell bioinks alongside Leydig cell-laden strands produces tubule-like geometries with interstitial spacing matching native testicular lobular architecture (Horvath-Pereira et al., 2023). Digital light processing approaches push resolution below 50 μm, allowing patterned placement of peritubular myoid cells around printed tubular cores and offering a level of architectural control likely to prove valuable for the next generation of standardized, reproducible organoid assays (Ravnic et al., 2017).

Because organoids are only one of several three-dimensional and dynamic culture options, it is helpful to position them against neighboring platforms. **Table 3** compares two-dimensional cultures, three-dimensional spheroids, testicular organoids, testicular explants, testis-on-chip, and multi-organ-chip systems across architecture, cellular composition, blood-testis barrier formation, spermatogenic maturation, scalability, and toxicological application, underscoring that no single platform is optimal across all criteria.

**Table 3:** Comparison of testicular *in vitro* and microphysiological model systems.

Model system	Architecture	Cellular composition	Blood-testis barrier	Spermatogenic maturation	Scalability	Toxicological application
2D monolayer culture	Flat single layer	Usually one cell type	None	None	Very high	High-throughput mechanistic screening
3D spheroid	Compact cell aggregate	One or few cell types	Minimal	Limited	High	Aggregate viability and steroidogenesis assays
Testicular organoid	Self-organized tubule-like	Multiple testicular lineages	Partial	Round to occasional elongated spermatid	Moderate	Multi-endpoint EDC and drug toxicity screening
Testicular explant	Native tissue fragment	Full native complement	Preserved short-term	Can complete (mainly rodent)	Low	Short-term mechanistic and proof-of-concept studies
Testis-on-chip	Perfused microfluidic device	Defined co-culture or embedded organoid	Inducible and measurable (TEER)	Partial	Low to moderate	Dynamic barrier toxicity and low-dose exposure
Multi-organ chip	Linked perfused compartments	Testis plus metabolic organ modules	Variable	Limited	Low	Metabolism-coupled systemic toxicity

### 3.3 Characterization and quality assessment of organoid maturation

Judging whether a testicular organoid has reached a biologically meaningful state of maturity remains one of the more difficult problems in the field, and the absence of consensus metrics has muddied cross-laboratory comparison for years. Structural maturity forms the first tier of evaluation. Hematoxylin-eosin sections reveal whether tubule-like structures exhibit concentric layering reminiscent of seminiferous epithelium, while immunohistochemistry for SOX9, VASA, and SYCP3 confirms the presence of Sertoli cells, germ cells, and meiotic figures respectively (Edmonds and Woodruff, 2020). Flow cytometric quantification of cellular composition—particularly the ratio of germ cells to somatic supports—offers a more objective structural readout than visual scoring alone, and deviations beyond roughly ±15% from native tissue proportions typically correlate with impaired downstream function ((Sakib et al., 2019a).

Functional maturity demands a different suite of assays. Testosterone secretion into conditioned media, measured by ELISA or liquid chromatography-mass spectrometry, serves as the canonical Leydig cell readout, with physiologically relevant output generally exceeding 1 ng/mL over 24 hours (Strandgaard et al., 2022). Meiotic progression can be tracked through SYCP3 staining combined with DAPI-based DNA content analysis, where successful pachytene entry is evidenced by synaptonemal complex elongation and the 4N peak shifting toward 1N as haploid cells emerge. Transcriptomic profiling of stage-specific markers—PLZF for undifferentiated spermatogonia, STRA8 for meiotic commitment, PRM1 for spermiogenesis—provides molecular granularity that morphology alone cannot resolve (Guo et al., 2021).

To reconcile these heterogeneous indicators, a composite quality score can be adopted. A weighted additive model, commonly applied in organoid quality control literature (Kim et al., 2020), takes the form.

(4) $$Q=\sum_{i=1}^{n}w_{i}\cdot S_{i}$$

where *S_i_* denotes the normalized score (0–1) for each indicator and *w_i_* its assigned weight. For situations where multiple endpoints must jointly exceed threshold values, a geometric mean formulation better penalizes bottleneck parameters:

(5) $$Q_{g}={\left(\prod_{i=1}^{n}S_{i}^{w_{i}}\right)}^{\frac{1}{\sum w_{i}}}$$

which follows standard composite indexing practice (Driehuis et al., 2020). **Table 4** consolidates the principal indicators considered essential for routine quality gating; the acceptance thresholds listed there are not proposed *de novo* but are adapted from values reported in the cited organoid and quality-control literature, and the supporting references are given in the final column.

**Table 4:** Evaluation indicator system for testicular organoid maturation.

Indicator domain	Specific metric	Detection method	Acceptance threshold	References
Morphology	Tubule-like structure formation	Bright-field microscopy, H&E	≥60% of constructs	(Edmonds and Woodruff, 2020)
Cellular composition	Sertoli/germ cell ratio	Flow cytometry	1:2 to 1:4	(Sakib et al., 2019a)
Barrier function	Occludin/ZO-1 localization	Immunofluorescence	Continuous apical staining	(Edmonds and Woodruff, 2020)
Steroidogenesis	Testosterone output	ELISA or LC-MS	>1 ng/mL per 24 h	(Strandgaard et al., 2022)
Meiotic entry	SYCP3-positive cells	Immunofluorescence	≥10% of germ cells	(Edmonds and Woodruff, 2020)
Haploid generation	1N DNA content fraction	Flow cytometry	≥2% of germ cells	(Sakib et al., 2019a)
Transcriptome fidelity	Stage-specific marker panel	qPCR or RNA-seq	Correlation r ≥ 0.7 with native	(Guo et al., 2021)
Viability	Caspase-3 negative fraction	Immunostaining	≥85%	(Kim et al., 2020)

As summarized in **Table 4**, no single metric suffices, and the integrated score derived from Equations 4 and 5 offers a pragmatic handle for comparing organoid batches across protocols and laboratories (Huch and Koo, 2015).

## 4 Applications of testicular organoids in male reproductive toxicology assessment

### 4.1 Toxicity evaluation of environmental endocrine-disrupting chemicals

Endocrine-disrupting chemicals have become the proving ground for testicular organoid toxicology, largely because their low-dose, non-monotonic behavior has long defied conventional assay logic. Bisphenol A and its structural analogs—BPS, BPF, and BPAF—produce distinctive signatures in organoid systems that rodent studies often miss. Exposure of human iPSC-derived testicular organoids to BPA at 10 nM, a concentration within the range of human urinary biomarkers, suppresses StAR transcription and lowers testosterone output by roughly 35% after 14 days, an effect that requires doses three orders of magnitude higher in TM3 Leydig monolayers (Cong et al., 2021). BPS, once marketed as a safer substitute, unexpectedly disrupts Sertoli tight junction assembly at 1 μM in decellularized matrix-based organoids, with occludin mislocalization preceding any overt viability decline (Li et al., 2022). Phthalate monoesters follow a different pattern: mono-(2-ethylhexyl) phthalate at 10 μM triggers germ cell apoptosis through oxidative stress and MAPK activation in three-dimensional cultures, while the parent diester remains largely inert—a bioactivation dependency that flat cultures lacking metabolic competence frequently underestimate (Wang et al., 2022). Polychlorinated biphenyls such as PCB153 accumulate preferentially in the lipid-rich Leydig compartment and suppress CYP17A1 expression over a 21-day exposure window, recapitulating endocrine patterns observed in occupationally exposed cohorts (Hu et al., 2021).

**Figure 3:**
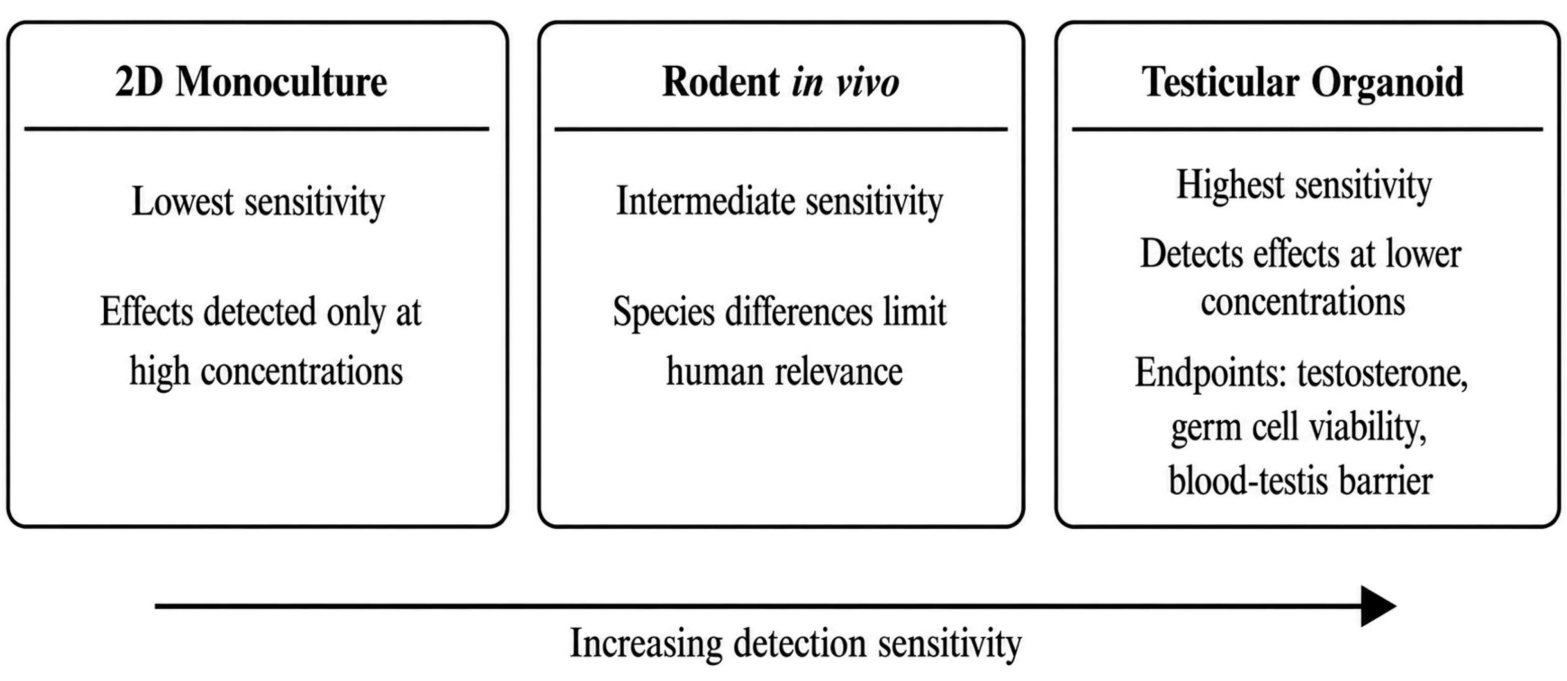
Conceptual comparison, prepared by the authors, of the relative sensitivity of two-dimensional monocultures, rodent *in vivo* models, and testicular organoids in detecting toxicity of bisphenol A, MEHP, BPS, and PCB153, for representative endpoints including testosterone output, germ cell viability, and blood-testis barrier integrity. The comparison is qualitative and is based on findings reported in the individual studies cited in this section (Cong et al., 2021; Hu et al., 2021; Richardson et al., 2021; Li et al., 2022; Wang et al., 2022; Sakib et al., 2019b); it summarizes relative sensitivity across platforms rather than a statistical meta-analysis, and no pooled error estimates are implied.

Comparative analyses across model platforms reveal a consistent sensitivity hierarchy. For BPA, the *EC*_50_ for testosterone suppression drops from approximately 12 μM in TM3 monocultures to 0.8 μM in Sertoli-Leydig co-cultures and further to 0.04 μM in tri-lineage organoids (Richardson et al., 2021). **Figure 3** presents comparative dose-response profiles for four representative endocrine disruptors across three model platforms.

The results depicted in **Figure 3** indicate that organoid platforms routinely detect effects at concentrations 10–100 fold below those required in monolayer systems, with the gap widening for compounds whose toxicity depends on intercellular crosstalk or three-dimensional barrier function (Sakib et al., 2019b).

Long-term low-dose exposure reveals perhaps the most distinctive organoid advantage. Continuous dosing with BPA at 1 nM over 60 days in human testicular organoids produces cumulative epigenetic drift, including hypomethylation of imprinted loci H19 and MEG3 in germ cell fractions, a phenotype inaccessible to short-duration assays (Santangeli et al., 2021). Such findings align with epidemiological signals linking chronic environmental BPA exposure to sperm quality decline and suggest that organoid endpoints may ultimately redefine what “adverse” means at environmentally relevant concentrations. **Table 5** summarizes the principal endocrine disruptors studied in testicular organoid systems alongside their characteristic effects and relative potencies. As **Table 5** shows, the spectrum of detectable endpoints spans steroidogenic, germinal, and junctional domains, reinforcing the point that multi-target readouts within a single organoid construct are needed to capture the full phenotypic reach of these compounds.

**Table 5:** Toxic effects of common endocrine-disrupting chemicals in testicular organoid models.

Compound	Effective concentration range	Principal cellular target	Characteristic endpoint	Exposure duration	References
Bisphenol A	1 nM – 10 μM	Leydig cells	Testosterone suppression, StAR downregulation	7–60 days	(Cong et al., 2021)
Bisphenol S	0.1–10 μM	Sertoli cells	Tight junction disassembly, occludin loss	14 days	(Li et al., 2022)
MEHP	1–50 μM	Germ cells	Apoptosis via MAPK and oxidative stress	7–21 days	(Wang et al., 2022)
DEHP (parent)	10–100 μM	Requires metabolic activation	Minimal direct effect in static culture	14 days	(Wang et al., 2022)
PCB153	0.1–5 μM	Leydig cells	CYP17A1 suppression, lipid accumulation	21 days	(Hu et al., 2021)
Parabens (methyl/propyl)	1–10 μM	Sertoli-germ interface	SSC niche disruption	14–28 days	(Joensen et al., 2020)
PBDE-47	0.5–5 μM	Multiple lineages	Oxidative damage, meiotic arrest	21 days	(Gore et al., 2020)

### 4.2 Reproductive toxicity screening of drugs and chemical agents

Pharmaceutical reproductive toxicity has emerged as a second major application domain where testicular organoids demonstrate clear diagnostic value, particularly for agents whose cellular targets span multiple testicular compartments. Alkylating chemotherapeutics such as cyclophosphamide and busulfan produce characteristic germ cell depletion patterns in human organoid cultures: exposure to 4-hydroperoxycyclophosphamide at 1 μM for 48 hours triggers preferential loss of differentiating spermatogonia while sparing the PLZF-positive stem cell pool, a selectivity that helps explain the partial fertility recovery observed in some patient populations (Easley et al., 2021). Cisplatin at 0.5–5 μM induces dose-dependent apoptosis through DNA adduct formation and p53 activation, with organoid *IC*_50_ values of approximately 1.8 μM aligning more closely with clinically reported testicular concentrations than the 8–12 μM range typical of monolayer Sertoli assays (Lee et al., 2021). Among antibiotics, gentamicin and nitrofurantoin elicit oxidative damage in organoid Leydig cells at therapeutically relevant concentrations, with nitrofurantoin producing a 45% decline in testosterone output at 20 μM over 10 days—a finding that corroborates clinical case reports of transient subfertility in treated males (Drobnis and Nangia, 2017). Immunosuppressants including cyclosporine A and sirolimus disrupt Sertoli-germ cell adhesion through dysregulation of the mTOR-autophagy axis, an effect traced by autophagosome accumulation and LC3-II immunoblotting in three-dimensional cultures (Xu et al., 2021).

High-throughput adaptation has advanced the platform’s screening utility. Miniaturized 384-well organoid arrays combined with automated fluorescence imaging and machine learning-based morphological scoring have enabled concurrent testing of several hundred compounds per run, with reported hit confirmation rates of the order of 80% when benchmarked against known testicular toxicants in individual studies (Carson et al., 2021); such automated scoring remains exploratory pending protocol standardization. Multi-omics integration layers additional resolution: simultaneous transcriptomic, proteomic, and metabolomic profiling of exposed organoids has uncovered mechanistic signatures distinguishing steroidogenic disruption from germotoxic injury, and single-cell RNA sequencing has mapped drug responses to specific cellular subpopulations that bulk assays blur together (Sohni et al., 2022).

Quantitative toxicity prediction rests on robust dose-response modeling. Applying a four-parameter logistic model (Motulsky and Christopoulos, 2004), drug-induced viability curves are described by Equation 6).

(6) $$V\left(C\right)=V_{min}+\frac{V_{max}-V_{min}}{1+{\left(\frac{C}{IC_{50}}\right)}^{h}}$$

where *V*(*C*) is the normalized viability at concentration *C*, *V_min_* and *V_max_* the lower and upper asymptotes, *IC*_50_ the half-maximal inhibitory concentration, and *h* the Hill slope (standard formulation). Fitted *IC*_50_ values then feed into a tiered hazard classification scheme, where compounds with *IC*_50_ below 1 μM are flagged as high-risk, 1–50 μM as moderate, and above 50 μM as low concern, calibrated against reference testicular toxicants.

**Figure 4:**
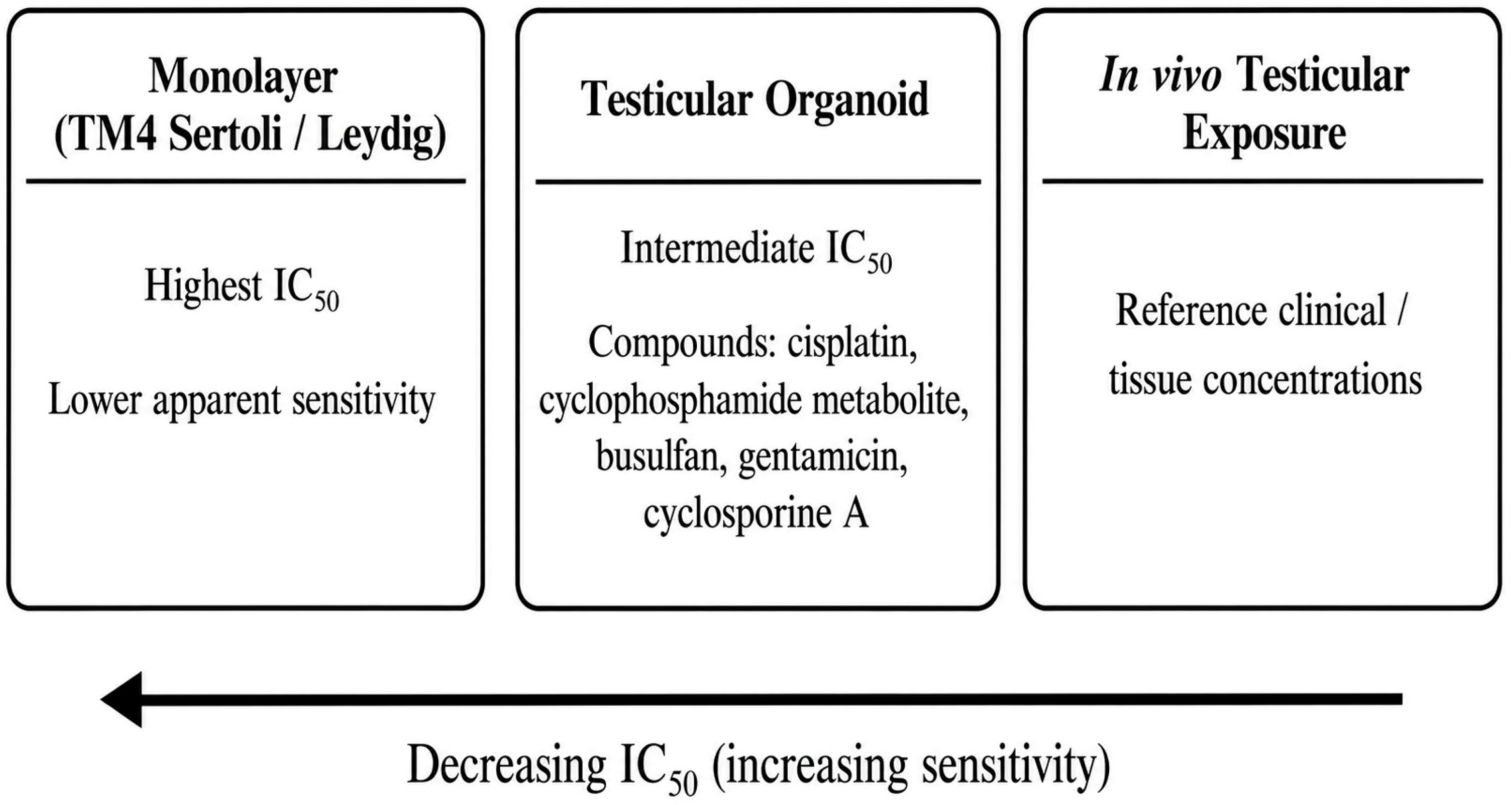
Conceptual comparison, prepared by the authors, of *IC*_50_ values for representative pharmaceuticals—cisplatin, cyclophosphamide metabolite, busulfan, gentamicin, and cyclosporine A—reported in.

As **Figure 4** indicates, organoid-derived *IC*_50_ values occupy an intermediate range between monolayer estimates and *in vivo* testicular exposure data, and this intermediate positioning is what makes the platform useful for bridging mechanistic and translational toxicology.

### 4.3 Mechanistic dissection of toxicity and biomarker discovery

Multi-omics integration has transformed testicular organoids from a phenotypic readout platform into a mechanistic discovery engine. Single-cell RNA sequencing of organoids exposed to cadmium chloride at 5 μM revealed cell-type-specific transcriptional reprogramming: Sertoli clusters exhibited early induction of metallothionein family genes and later collapse of junction-associated transcripts, whereas spermatogonia showed activation of p53 target genes prior to apoptotic clearance—temporal ordering that bulk sequencing simply cannot resolve (Wang et al., 2022). Proteomic analysis via data-independent acquisition mass spectrometry has identified post-translational modifications that escape transcriptomic detection, such as oxidative carbonylation of testicular peroxiredoxin 4 following tert-butyl hydroperoxide exposure, positioning this protein as an early sentinel of redox imbalance (Shi et al., 2021). Metabolomic profiling rounds out the picture, with untargeted LC-MS detecting shifts in carnitine shuttle intermediates and lactate-to-pyruvate ratios that precede overt cell death by 48 hours in phthalate-treated constructs (Tan et al., 2022).

Three mechanistic pathways recur across these datasets. Oxidative stress, mediated by NADPH oxidase activation and glutathione depletion, converges on lipid peroxidation products detectable through 4-HNE immunostaining and MDA quantification (Aitken and De Iuliis, 2020). Mitochondrial dysfunction appears as a secondary but amplifying hit, with Seahorse extracellular flux analysis revealing depressed oxygen consumption rates and uncoupled respiration in Leydig cells exposed to bisphenol analogs or mitochondrially targeted drugs (Liu et al., 2021). Epigenetic remodeling constitutes a third axis: reduced representation bisulfite sequencing of organoids exposed chronically to low-dose BPA has mapped hypomethylation across imprinted loci and germline-specific promoters, alterations that persist even after toxicant withdrawal and hint at transgenerational consequences invisible to acute assays (Mao et al., 2021).

**Figure 5:**
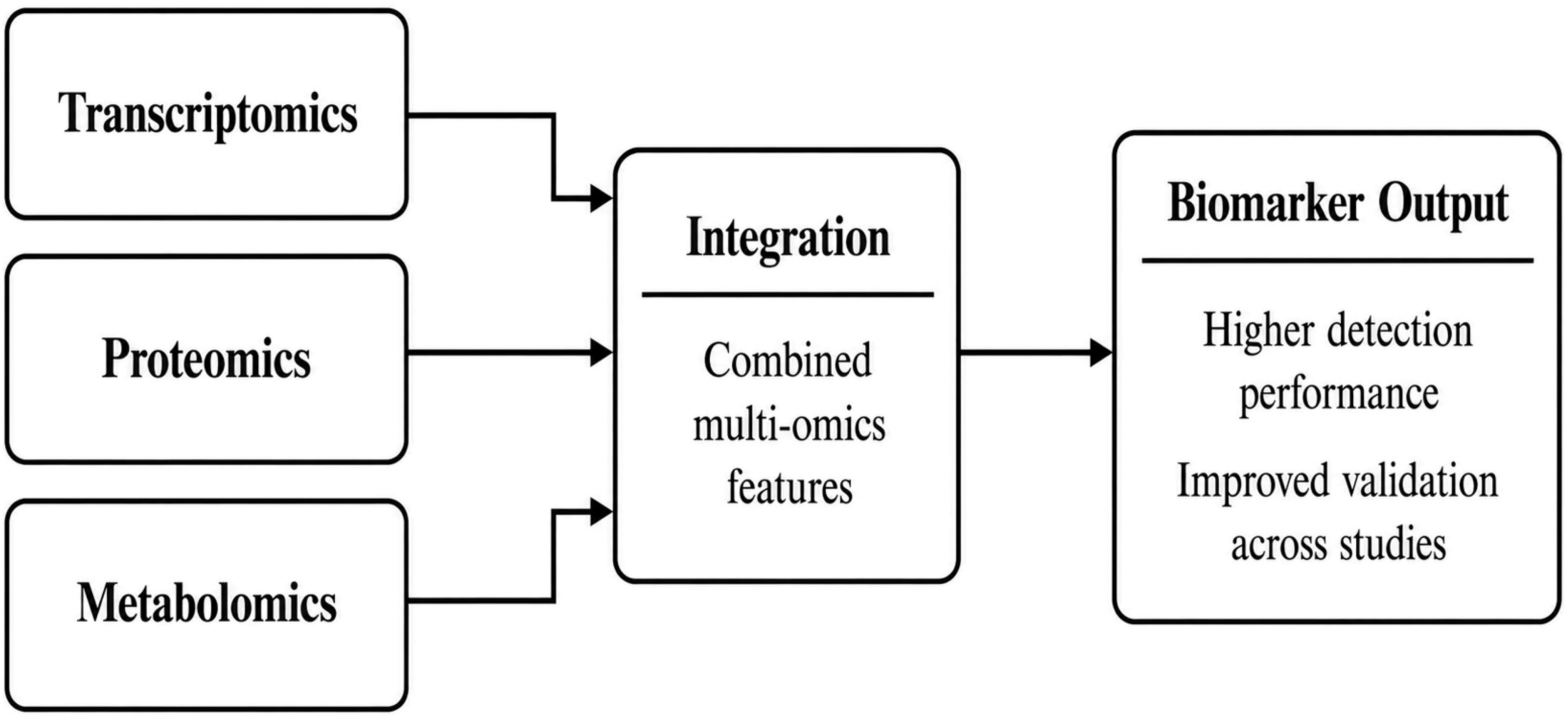
Conceptual comparison, prepared by the authors, of single-omics versus integrated multi-omics approaches for reproductive toxicity biomarker discovery in testicular organoids, across transcriptomic, proteomic, metabolomic, and combined analytical pipelines. The figure illustrates qualitative trends in detection performance and validation success synthesized from the cited studies (Luechtefeld et al., 2016; Aitken and De Iuliis, 2020; Liu et al., 2021; Mao et al., 2021; Shi et al., 2021; Jambor et al., 2022; Sohni et al., 2022; Tan et al., 2022; Wang et al., 2022) rather than a quantitative meta-analysis, and no pooled performance statistics are implied.

Machine learning has been applied to biomarker prioritization from these high-dimensional datasets. Random forest classifiers trained on integrated transcriptomic and proteomic features have been reported to reach area-under-curve values of around 0.89 for distinguishing germotoxic from non-germotoxic mechanisms, outperforming single-omics models in the studies examined (Luechtefeld et al., 2016). These computational approaches remain exploratory, however: because organoid construction is not yet standardized, model performance is difficult to compare across laboratories and should be regarded as preliminary rather than validated. **Figure 5** compares biomarker identification efficiency across progressively integrated analytical layers.

As **Figure 5** illustrates, integrated pipelines tend to outperform individual omics layers in the reported studies, and the marginal gain from adding metabolomics to transcriptomic-proteomic combinations appears most pronounced for low-dose exposures. **Table 6** consolidates the most robust candidate biomarkers emerging from these analyses.

**Table 6:** Candidate male reproductive toxicity biomarkers identified through testicular organoid studies.

Biomarker	Biological source	Associated toxicity pathway	Validation status	References
INSL3	Leydig cell secretome	Steroidogenic disruption	Clinically correlated	(Jambor et al., 2022)
Inhibin B	Sertoli cell secretome	Sertoli dysfunction	Clinically validated	(Strandgaard et al., 2022)
4-HNE protein adducts	Germ cell proteome	Oxidative damage	Preclinical validation	(Shi et al., 2021)
microRNA-34c	Germ cell-derived exosomes	Spermatogenic arrest	Human cohort tested	(Jambor et al., 2022)
Peroxiredoxin 4 carbonylation	Testicular proteome	Redox imbalance	Preclinical only	(Shi et al., 2021)
H19 methylation status	Germ cell epigenome	Imprinting disturbance	Exploratory	(Mao et al., 2021)
Lactate/pyruvate ratio	Culture metabolome	Sertoli metabolic shift	Preclinical only	(Tan et al., 2022)
Connexin 43 phosphorylation	Sertoli junctional proteome	BTB disruption	Preclinical validation	(Xu et al., 2021)

As **Table 6** lists, candidate markers span secreted factors amenable to clinical translation and intracellular signatures useful mainly for mechanistic confirmation, and coordinated validation across these tiers is likely to define the next phase of organoid-enabled biomarker research (Jambor et al., 2022).

## 5 Discussion

Taken together, the evidence assembled across construction strategies and application domains paints a picture of testicular organoids as a technology whose strengths and limitations are both substantial and inseparable. The advantages relative to conventional assessment systems are concrete rather than rhetorical. Human-derived organoids sidestep the interspecies gap that has confounded rodent extrapolation for decades, delivering dose-response profiles for compounds like phthalate monoesters and bisphenol analogs at concentrations reported in several studies to be one to two orders of magnitude below monolayer detection limits (Richardson et al., 2021; Wang et al., 2022). The preserved three-dimensional architecture permits paracrine crosstalk among Sertoli, Leydig, and germ cells to manifest in ways that flat cultures structurally cannot replicate (Baert et al., 2017). Throughput, while still below that of biochemical screens, now exceeds what rodent bioassays can reasonably deliver, and the mechanistic resolution afforded by single-cell multi-omics applied to these systems opens investigational depth previously unavailable (Sohni et al., 2022).

Several bottlenecks nonetheless remain serious and, in some cases, underappreciated. Blood-testis barrier reconstitution in most organoid protocols is partial at best—transepithelial resistance values typically plateau well below physiological levels, and apical-basal polarity often degrades after three to four weeks (Park et al., 2021). Long-term culture stability beyond 60 days is rarely achieved without progressive loss of germ cell complements, constraining chronic exposure studies to timeframes shorter than a full spermatogenic cycle. Inter-donor heterogeneity introduces variability that complicates reproducibility, and elongated spermatozoa formation remains the exception rather than the rule, restricting the platform’s reach into late spermiogenic toxicity. These shortfalls are consequential because many regulatory endpoints depend precisely on the late stages of spermatogenesis that current models reach least reliably. The barriers to complete maturation are multifactorial: culture medium composition and the balance of endocrine support (FSH, hCG, and retinoic acid timing), the identity and stiffness of the extracellular matrix, and physicochemical parameters such as temperature and oxygenation all shape how far differentiation proceeds (Kanatsu-Shinohara and Shinohara, 2013; Alves-Lopes et al., 2017; Vermeulen et al., 2018; Richer et al., 2020). Equally important are the absence of a vascular supply, which limits nutrient and oxygen delivery to organoid cores, incomplete recapitulation of native tubular architecture, and culture durations that fall short of the roughly 74-day human spermatogenic cycle. A further, cross-cutting limitation is that construction protocols differ widely between laboratories in cell ratios, matrix, hormonal supplementation, and duration, which makes direct quantitative comparison across studies difficult; the present review therefore synthesizes trends qualitatively and treats reported quantitative values as study-specific rather than pooled.

Convergence with adjacent technologies offers realistic paths forward. Coupling organoids with microfluidic organ-on-chip architectures improves nutrient perfusion and enables connected metabolism from hepatic modules, while CRISPR-based genetic engineering of donor stem cells allows targeted dissection of susceptibility genes. Artificial intelligence-driven image analysis and predictive modeling promise to translate morphological heterogeneity into quantitative toxicity signatures rather than treating it as noise to be averaged away. Each of these integrations carries its own validation burden, which is easy to underestimate.

Regulatory acceptance presents perhaps the most demanding challenge. Standardization of cell sources, matrix formulations, culture durations, and endpoint definitions remains fragmented across laboratories, and formal inter-laboratory validation studies of the kind that secured OECD acceptance for earlier alternative methods are only beginning (Balls, 2020). Meaningful progress will require agreed standard operating procedures, demonstrated inter-laboratory reproducibility, and a defined panel of positive and negative reference compounds against which assay performance can be calibrated, followed by multicenter validation under recognized New Approach Methodology frameworks. Extrapolation from organoid dose-response data to human population risk requires explicit handling of *in vitro*–to–*in vivo* (IVIVE) uncertainty factors, donor representativeness, and the mismatch between static culture concentrations and dynamic human exposures, particularly for the low-dose and mixture scenarios that dominate real-world exposure (Luechtefeld et al., 2016; Drakvik et al., 2020). Until these elements are in place, organoid data are better positioned to support weight-of-evidence and mechanistic read-across than to serve as stand-alone replacements within formal risk assessment. Productization and clinical translation will further demand sustained collaboration among developmental biologists, toxicologists, bioengineers, statisticians, and regulators—a coalition whose absence has arguably slowed progress as much as any single technical barrier.

## 6 Conclusion

This review has mapped the construction strategies, evaluation applications, and persistent technical bottlenecks of testicular organoid models within the landscape of male reproductive toxicology. By synthesizing cellular sourcing approaches, matrix and platform choices, maturation benchmarks, and deployment across endocrine disruptor screening, pharmaceutical toxicity testing, and mechanistic biomarker discovery, this review suggests that organoid technology occupies a distinct methodological niche that neither rodent bioassays nor conventional two-dimensional cultures can adequately fill. Its core value lies in sharpening predictive accuracy at human-relevant exposure concentrations, reducing reliance on whole-animal testing in accordance with 3Rs principles, and enabling mechanistic resolution compatible with the ambitions of precision toxicology. At the same time, these models are not yet mature or sufficiently validated to broadly replace *in vivo* testing, and their present role is best understood as complementary.

The principal contribution offered here is the integration of morphological, functional, molecular, and epigenetic readouts into a unified evaluation framework that may support construction of a next-generation assessment system for male reproductive hazards. Practical implications extend to pharmaceutical safety evaluation, environmental risk management, and protection of reproductive health in populations facing complex chemical exposures. Two main limitations of this synthesis should be acknowledged: the breadth of data integrated across heterogeneous studies inevitably sacrifices some quantitative precision, and head-to-head comparisons of organoid platforms against identical reference compound panels remain sparse.

Future priorities include codifying standardized construction protocols to enable cross-laboratory reproducibility, developing automated high-throughput screening architectures compatible with industrial workflows, integrating multi-organ-on-chip couplings to capture systemic metabolism and feedback, and engaging regulatory bodies early in validation dialogues. Progress on these fronts will determine whether testicular organoids mature from a promising research model into a validated, transformative tool for safeguarding male reproductive health.

## Data Availability

All data generated and analyzed during the current study are available from the corresponding author upon reasonable request.

## Glossary

BPA: Bisphenol A
BPS: Bisphenol S
BPF: Bisphenol F
BPAF: Bisphenol AF
BTB: Blood-Testis Barrier
CYP17A1: Cytochrome P450 17A1
DEHP: Di(2-ethylhexyl) Phthalate
*EC*_50_: Half-Maximal Effective Concentration
EDC: Endocrine-Disrupting Chemical
ELISA: Enzyme-Linked Immunosorbent Assay
EPA: Environmental Protection Agency
FGF2: Fibroblast Growth Factor 2
FSH: Follicle-Stimulating Hormone
GDNF: Glial Cell Line-Derived Neurotrophic Factor
GFRα1: GDNF Family Receptor Alpha 1
GnRH: Gonadotropin-Releasing Hormone
hCG: Human Chorionic Gonadotropin
H&E: Hematoxylin and Eosin
4-HNE: 4-Hydroxynonenal
*IC*_50_: Half-Maximal Inhibitory Concentration
ID4: Inhibitor of DNA Binding 4
INSL3: Insulin-Like Factor 3
iPSC: Induced Pluripotent Stem Cell
LC-MS: Liquid Chromatography-Mass Spectrometry
LH: Luteinizing Hormone
MAPK: Mitogen-Activated Protein Kinase
MDA: Malondialdehyde
MEHP: Mono-(2-ethylhexyl) Phthalate
mTOR: Mechanistic Target of Rapamycin
NAM: New Approach Methodology
OECD: Organization for Economic Co-operation and Development
PBDE: Polybrominated Diphenyl Ether
PCB: Polychlorinated Biphenyl
PDMS: Polydimethylsiloxane
PFAS: Per- and Polyfluoroalkyl Substances
PGCLC: Primordial Germ Cell-Like Cell
PLZF: Promyelocytic Leukemia Zinc Finger
PRM1: Protamine 1
qPCR: Quantitative Polymerase Chain Reaction
REACH: Registration, Evaluation, Authorization and Restriction of Chemicals
RNA-seq: RNA Sequencing
SCF: Stem Cell Factor
SOX9: SRY-Box Transcription Factor 9
SSC: Spermatogonial Stem Cell
StAR: Steroidogenic Acute Regulatory Protein
STRA8: Stimulated by Retinoic Acid 8
SYCP3: Synaptonemal Complex Protein 3
TEER: Transepithelial Electrical Resistance
TG: Test Guideline
VASA: DEAD-Box Helicase 4
WT1: Wilms Tumor 1
ZO-1: Zonula Occludens-1
α-SMA: Alpha Smooth Muscle Actin
